# The Chinese Version of the Intuitive Eating Scale‐2 Adapted for Pregnant Women: Psychometric Properties and Associations With Diet Quality

**DOI:** 10.1002/brb3.70568

**Published:** 2025-05-30

**Authors:** Xingyi Jin, Jian Zhu, Da Pan, Lingzhen Sun, Rui Wang, Niannian Wang, Jiongnan Wang, Chunyan Yuan, Shaokang Wang, Guiju Sun

**Affiliations:** ^1^ Key Laboratory of Environmental Medicine and Engineering of Ministry of Education, Department of Nutrition and Food Hygiene, School of Public Health Southeast University Nanjing China; ^2^ Danyang Maternal and Child Health Hospital, Danyang Zhenjiang China; ^3^ Department of Gynecology and Obstetrics Zhongda Hospital Southeast University Nanjing China; ^4^ Clinical Medical Research Center for Plateau Gastroenterological Disease of Xizang Autonomous Region and School of Medicine Xizang Minzu University Xianyang China

**Keywords:** dietary quality, eating behaviors intuitive eating, pregnancy, psychometric properties

## Abstract

**Background:**

Pregnancy is a special period that is strongly influenced by dietary interventions, and many pregnant women develop gestational diabetes caused by conditions such as poor diet. Eating behavior interventions for women during this period are therefore very important, but unfortunately, there is a lack of established studies on intuitive eating in pregnant women.

**Methods:**

This study examined the psychometric properties of a Chinese version of the IES‐2 scale adopted in a group of pregnant women, as well as the relationship between intuitive eating characteristics and dietary quality. A total of 581 pregnant women completed the study which included the Intuitive Eating Scale (IES‐2), the Depressive Symptom Scale (EPDS), the Anxiety Symptom Scale (SAS), the Parenthood Stress Scale (PPS), and The Dietary Guidelines Adherence Index for Pregnant Women during Pregnancy (CDGCI‐PW).

**Results:**

The results show that the modified scales have good quality in the Chinese pregnant women population (CMIN/DF = 1.756, CFI = 0.925, TLI = 0.909, RMSEA = 0.037) and that the scale scores are correlated with depression and anxiety of pregnant women and correlated with overall diet quality during pregnancy.

**Conclusions:**

The final six factors (avoiding forbidden foods, avoiding emotional eating, body.food choice congruence, avoiding food‐related coping strategies, permission to eat, and reliance on hunger and satiety cues) structure of the revised IES‐2 was confirmed. Moreover, the higher the intuitive diet, the better the quality of the pregnant woman's diet will be. This scale can further assess eating behaviors in different pregnancy states within the Chinese pregnant women population.

## Graphical abstract

The modified scales have good quality in the Chinese pregnant women population, and the scale scores are correlated with depression and anxiety of pregnant women and correlated with overall diet quality during pregnancy. Moreover, the higher the intuitive diet, the better the quality of the pregnant woman's diet will be.

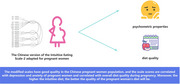

## 1 Introduction

Intuitive eating involves relying on one's own physiologic hunger and satiety cues instead of relying on situational or emotional cues to guide eating behavior (Tribole 2003, 2006; Tylka 2006). It is based on the assumption that individuals are intrinsically capable of self‐regulating their food intake when they can distinguish between physical hunger and emotional hunger (Tribole 2003). Intuitive eating is associated with lower body mass index, improved mental health conditions, and healthy diet quality (Bruce and Ricciardelli 2016; Hawley et al. 2008; Gast et al. 2015). Researchers found that eating behaviors relating to food selection, timing, and quantity affect dietary quality and weight in non‐pregnant samples (Małachowska and Jeżewska‐Zychowicz 2022). There were numerous studies linking pre‐pregnancy maladaptive eating behaviors (e.g., restraint, disordered eating) to excess and inadequate gestational weight gain (GWG) as well as poor dietary intake during pregnancy (Conway et al. 1999; Mumford et al. 2008). Poor eating behaviors during pregnancy are associated with adverse pregnancy outcomes (e.g., gestational diabetes, macrosomia). Therefore, monitoring eating behaviors during pregnancy is important to promote a healthy pregnancy.

The Intuitive Eating Scale (IES) was developed and validated by Tylka et al. in 2006, and a revised version was published in 2013 (Intuitive Eating Scale‐2 [IES‐2]) (Tylka and Kroon Van Diest 2013). There is a high level of convergent and discriminant validity, test‐retest reliability, and factor structure stability for the IES‐2 (Tylka and Kroon Van Diest 2013; Van Dyke and Drinkwater 2014). The IES‐2 is a 23‐item instrument that was found to fit a four‐dimensional factor structure. The four dimensions reflect eating for physical rather than emotional reasons (i.e., Rather than eating when one is emotionally distressed, one should eat when one is physically hungry: eight items), unconditional permission to eat (i.e., The willingness of an individual to consume certain foods when he or she is hungry and a refusal to label food as forbidden: six items), reliance on hunger and satiety cues (i.e., A person's reliance on his or her internal hunger and satiety cues to guide eating habits: six items), and body–food choice congruence (i.e., A tendency to make food choices that are in accordance with one's health and the functioning of the body: three items). There were validations for the IES‐2 questionnaire in multiple languages for use in China, France, and Germany, as well as other populations in certain countries (Camilleri et al. 2015; Ji et al. 2024; Ruzanska and Warschburger 2017). However, the current study found that the factor structure of the scale is inconsistent across populations. For instance, a five‐factor structure can be observed in the Mexican population of pregnant women (Flores‐Quijano et al. 2023). A study of a low‐income population in the United States suggested that the scale could be divided into six factors, while studies in Germany, Portugal, and France suggested that it could be divided into three factors. Additionally, the scale entries have also been deleted to varying degrees (Carbonneau et al. 2016; Dockendorff et al. 2012; Khalsa et al. 2019; Warren et al. 2017; van Dyck et al. 2016). Beyond dimensionality, other psychometric indices of the IES‐2 are generally well supported in diverse national groups. More intuitive eating has been associated with lower depressive symptoms, lower anxiety, lower stress, and healthier food intake (Camilleri et al. 2015; Carbonneau et al. 2017; Ji et al. 2024; König et al. 2021; Tylka and Kroon van Diest 2013). Nevertheless, previous studies have rarely observed an association between overall diet quality and intuitive eating, and in recent years, studies have begun to demonstrate that intuitive eating behavior in childhood is associated with a better overall diet quality in adulthood (Małachowska et al. 2023). It should be noted that due to the unique nature of pregnancy, the quality of the diet during this time of the pregnancy has a substantial impact not only on the health of the mother but also on the growth of her unborn child. It is therefore important to identify the factors that determine the quality of the nutrition consumed by a mother during her pregnancy.

During pregnancy, pregnant women experience increased stress and significant hormonal fluctuations. Several studies examined the psychometric properties of the first version of the IES in pregnant women (Ledoux et al. 2021; Lee et al. 2020; Paterson et al. 2019), and others assessed their psychometric properties in this population (Daundasekara et al. 2017; Paterson et al. 2018). The IES‐2 has also been applied during pregnancy (Quansah et al. 2019; Plante et al. 2019). According to Peterson and others, women tend to score higher on the dependent hunger and satiety signals subscale during pregnancy than they do pre‐pregnancy, indicating an increased awareness of perceived signals of hunger and satiety during pregnancy (Paterson et al. 2019). Ledoux and some other researchers found that during the course of pregnancy, a one‐point increase in the subscale score associated with eating for physical rather than emotional reasons resulted in an average weight loss of 2.72 kg. Further, for every one‐point increase in the IES score, the average weight gain during pregnancy decreased by 1.7 kg (Ledoux et al. 2021). As far as we are aware, neither the Chinese version of the IES‐2 nor its psychological properties have been tested on a group of pregnant women. Few previous studies also made significant distinctions between pregnancy stages.

It is therefore necessary to validate the Chinese version of IES‐2 in a sample of pregnant women in order to improve our understanding of pregnant women's intuitive eating behavior. This study (Tribole 2003) aimed at modifying the Chinese version of the IES‐2 based on pregnant women's characteristics (Tribole 2006), evaluating the psychometric properties of the Chinese version of the IES‐2, and (Tylka 2006) determining whether intuitive eating characteristics were associated with diet quality during pregnancy.

## 2 Materials and Methods

### 2.1 Study Design

Danyang Maternal and Child Health Hospital in Zhenjiang City, Jiangsu Province, China, was the location of the study, which ran from October 20, 2022, to September 3, 2023. This project involved 645 participants. As a result of excluding 64 participants due to lost visits, questionnaire failures, automatic withdrawals, and miscarriage, 581 pregnant women were included in this study's analysis after completing a baseline survey in early pregnancy and a mid‐pregnancy follow‐up survey. In the Danyang Maternal and Child Health Hospital, pregnant women were asked to complete a baseline survey within 12 weeks and to return to the Maternal and Child Health Hospital for follow‐up within 23–25 weeks. In order to ensure participant compliance, the researcher contacted each pregnant woman at least once by telephone and SMS message. Inclusion criteria were voluntary participation in the study and signing of an informed consent form, expecting to give birth at Danyang Maternal and Child Health Hospital, knowing Mandarin for barrier‐free communication, and understanding and correctly filling out the questionnaire. The following conditions were specifically excluded: being younger than 18 years of age and being diagnosed with AIDS, syphilis, heart disease, metabolic disorders, mood disorders, and other diseases that affected the study's purpose. The Ethics Committee of Danyang Maternal and Child Health Hospital gave its approval to the study. A specific flowchart can be found in Figure S1.

### 2.2 Basic Information Questionnaire

All participants completed a questionnaire, including age, week of gestation, constipation, Nutritional Guidance in Early Pregnancy, and Eating Behavior Change in Mid‐Pregnancy. There was some information about the hospital's electronic records, which included the sociodemographic data. The concept of nutritional guidance in early pregnancy refers to whether or not the pregnant woman has received professional nutritional supplements, dietary intake guidance, or a customized diet during pregnancy. The term “middle‐pregnancy diseases” refers to illnesses recorded in the hospital's electronic case file. It does not include diseases that existed before pregnancy, but rather new illnesses that developed after pregnancy. The following disorders are included but are not limited to metabolic disorders of pregnancy (gestational diabetes), thyroid disorders, high blood pressure, complications of pregnancy, and so forth. All participants signed an informed consent form.

### 2.3 Intuitive Eating

Intuitive eating is a construct believed to be composed of four components, including unconditional permission to eat (UPE), eating for physical rather than emotional reasons (EPR), reliance on hunger, and satiety cues to determine when and how much to eat (RHS) and body–food choice congruence. The number of items in UPE, EPR, RHS, and body–food choice congruence is six, eight, six, and three, respectively. There are 23 items on the IES‐2, which are rated on a five‐point Likert scale between “strongly disagree” and “strongly agree” (Tylka and Kroon van Diest 2013). Six of the items have been reverse coded. The average score for the entire scale and its four subscales should be calculated based on the sum of the items.

In order to conduct this study, we revised the scale based on the approved questionnaire in accordance with the specifics of the pregnant women group in terms of the relevant questions, and we used the Chinese version of the Intuitive Eating Scale‐2 which was verified in a Chinese population (Wu et al. 2022).

Prior to the commencement of the official test, we randomly invited 20 pregnant women who were being examined in the obstetrics department to participate in a pretest. We collected information on the gestational age, gestation period, weight, and others of each participant after determining that they met the inclusion and exclusion criteria for this study. A nutritionist consultation room within the hospital was set aside for a researcher (J.Z., Obstetrician, and Nutritionist) to conduct the interviews. About 20–40 min were spent interviewing the participants. We asked participants to complete the IES‐2 as well as a semi‐structured interview on eating behavior using the think aloud technique. A pregnant woman described to the researcher her perceptions of each item in the IES‐2 scare as well as what she found confusing or ambiguous.

Some of the feedbacks from these pregnant women are as follows:

‐ “I don't want to eat at all when I am suffering from pregnancy sickness, but my mother‐in‐law said that I have to ensure nutrition, thus, I dare not to completely rely on my body's signals”;
‐ “Obviously, I am not hungry, but I am worried about the baby's development, so I can only force myself to eat according to the plan”;
‐ “Because of frequent pregnancy sickness, I avoid eating though I feel hungry”;
‐ “I can pretty much just drink some water and eat a little bit of thin rice because when I eat, I vomit, and I don't care if I'm hungry or not anymore, so I can't understand it.”

In light of the semantic differences and special physiological conditions of pregnant women (e.g., loss of appetite caused by the reaction to pregnancy vomiting), the relevant questions were revised again, as indicated in items 19 and 20. The final Chinese version of the scale was culturally validated by four researchers in the fields of dietetics, clinical medicine, and psychology. See Table 1 for specific modification scales. The modified scale has been back‐translated from Chinese to English and is included in Table 1 in order to make it understandable to international readers.

**TABLE 1:** Various versions of the IES‐2 scale items.

IES‐2 original version	Revised English version of the IES‐2 (simplified Chinese version) for pregnancy	Modified pregnancy IES‐2 (simplified Chinese version)
Factor 1: Eating for physical rather than emotional reasons (EPR)		
1. I find myself eating when I'm feeling emotional, even when I'm not physically hungry.	During pregnancy, I find that when I am emotional, I go for food (even if it doesn't feel like I was physically hungry).	在怀孕期间, 我发现当我情绪激动时, 我就会去吃东西(即使我感觉身体并不饿)。
2. I find myself eating when I am lonely, even when I am not physically hungry.	During pregnancy, I find that when I felt lonely, I would go for food (even if I am not physically hungry).	在孕期, 我发现当我感到孤独时, 会去吃东西(即使身体并不饿)。
3. I use food to help me soothe my negative emotions.	During pregnancy, I use food to help me soothe my negative emotions.	在孕期, 我可以用食物去抚平我的消极情绪。
4. I find myself eating when I am stressed out, even when I'm not physically hungry.	During pregnancy, I find that when I feel stressed, I go for food (even if I am not feeling hungry out of my body).	在孕期, 我发现当我感受到压力的时候, 我就会去吃东西(即使我不是出于身体感到饿)。
5. I am able to cope with my negative emotions without turning to food for comfort.	During pregnancy, I am able to soothe my negative emotions. Negative feelings that I can soothe without having to eat my way through.	在孕期, 我有能力去抚平我的消极情绪。我可以不用通过吃东西的方式去安抚的消极情绪。
6. When I am bored, I do not eat just for something to do.	During pregnancy, when I am bored, I don't choose to get something to eat because I want something to do to pass the time.	在孕期, 当我感到无聊时, 我不会因为想找点事情去做打发时间而选择去吃点东西。
7. When I am lonely, I do not turn to food for comfort.	During pregnancy, when I feel lonely, I don't comfort myself by choosing to eat food at that point.	在孕期, 当我感到孤独的时候, 我不会在此时选择吃食物而逃避孤独。
8. I find other ways to cope with stress and anxiety than by eating.	During pregnancy, I find ways to cope with my stress and anxiety other than eating.	在孕期, 我找到了除了吃东西以外的方法去应对我的压力和焦虑。
Factor 2: Unconditional permission to eat (UPE)		
9. I try to avoid certain foods high in fat, carbohydrates, or calories.	During pregnancy, I try to avoid certain foods high in fat, carbohydrates, or calories.	怀孕期间, 我尽量避免食用某些高脂肪、高碳水化合物或高热量的食物。
10. If I am craving a certain food, I allow myself to have it.	During pregnancy, if I have a sudden craving for a certain food, I go for it.	怀孕期间, 如果我突然想吃某种食物, 我就会去吃。
11. I get mad at myself for eating something unhealthy.	During pregnancy, I get mad at myself for eating something unhealthy.	在孕期, 我发现我会因为吃不健康的东西而生气。
12. I have forbidden foods that I don't allow myself to eat.	During pregnancy, I clearly know that there are foods that I am not allowed to eat.	在孕期, 我明确知道我有不可以吃的食物。
13. I allow myself to eat what food I desire at the moment.	During pregnancy, I allow myself to eat what I want.	在孕期, 我会允许自己吃自己想吃的东西。
14. I do not follow eating rules or diet plans that dictate what, when, and/or how much to eat.	During my pregnancy, I do not have any dietary rules or plans. Even if I have I will not follow them (including what, when and how much to eat).	在孕期, 我没有任何的饮食规则和计划。即使有我也不会遵守。(包括吃什么、什么时候吃和吃多少)
Factor 3: Reliance on hunger and satiety cues (RHS)		
15. I trust my body to tell me when to eat.	During pregnancy, I think my body will tell me when I need to go for food.	在孕期, 我认为我的身体会告诉我什么时候要去吃东西
16. I trust my body to tell me what to eat.	During pregnancy, I think my body will tell me what I need to go for food.	在孕期, 我认为我的身体会告诉我需要去吃什么东西
17. I trust my body to tell me how much to eat.	During pregnancy, I think my body will tell me how much to eat.	在孕期, 我认为我的身体会告诉我需要吃的食物的量。
18. I rely on my hunger signals to tell me when to eat.	During pregnancy, I rely heavily on whether or not my body feels hunger signals to choose when to eat.	在孕期, 我主要依靠身体是否感到饥饿信号而选择什么时候进食。
19. I rely on my fullness signals to tell me when to stop eating.	During my pregnancy, I rely heavily on whether or not I feel full to choose when to stop eating.	在孕期, 我主要依靠是否感到饱腹感来选择何时停止进食。
20. I trust my body to tell me when to stop eating.	During pregnancy, I trust my body to tell me when to stop eating.	在孕期, 我相信我的身体会告诉我何时停止进食。
Factor 4: Body–food choice congruence (BFC)		
21. Most of the time, I desire to eat nutritious foods.	During pregnancy, I want to eat nutritious food.	在孕期的大多数时候, 我都希望吃有营养的食物。
22. I mostly eat foods that make my body perform efficiently.	During my pregnancy, I mainly eat foods that will make me feel like I am energizing my body.	在孕期, 我主要吃那些会让我觉得让我的身体有活力/高效的食物。
23. I mostly eat foods that give my body energy and stamina.	During pregnancy, I eat mostly foods that provide my body with energy and stamina.	在孕期, 我主要吃那些能为身体提供能量和体力的食物。

### 2.4 Depression

We utilized the total score of the Edinburgh Postnatal Depression Scale (EPDS) as a continuous measure of maternal depressive symptoms (Cox et al. 1987). The Edinburgh Postnatal Depression Scale was initially created to assess postnatal depression in women who had recently given birth. It was possible to measure the risk of prenatal depression using this scale which had been verified effectively. In this scale, 10 items were rated on a four‐point scale based on self‐report. The items were rated on a scale of 0–3 (0 = almost always, 3 = never, not at all). When the items were scored in a way, the negative statements would be reversed. Depression scores were higher when the total score was higher. A total score of more than 13 would indicate the presence of depression.

### 2.5 Anxiety

The widely used Zung's Self‐rating Anxiety Scale (SAS) was used to assess anxiety symptoms (Zung 1965). As a continuous measure of anxiety status, we used the total self‐reported SAS score of pregnant women in the present study. Each of the 20 items on the scale was rated on a four‐point scale (sometimes, some of the time, most of the time), with each of them rated on a four‐point scale (sometimes, some of the times, most of the time). There were some items with positive scores, and there were some items with negative scores, with a score range of 20–80. A participant would be identified as having anxiety disorder if the last score was greater than 50.

### 2.6 Pregnancy Stress

The Pregnancy Stress Scale (PPS), developed by Chan Changhua and some other researchers in the 1990s, was used to analyze the level and source of stress (C. Chen et al. 1989). To measure the stress status of pregnant women, we used the total PPS score. There were 30 items on the scale, each scored on a four‐point scale from 0 to 3, and the total score was the sum of the 30 scores. When the total score on the scale was higher, it would indicate that the pregnancy was more stressful.

### 2.7 Pregnancy Dietary Quality

Based on the dietary guidelines for Chinese residents (2016), the Dietary Guidelines Adherence Index for Pregnant Women during Pregnancy (CDGCI‐PW) was a clinical evaluation tool to determine and evaluate the dietary quality of expectant mothers. To assess the overall dietary quality of pregnant women, we used the CDGCI‐PW score. This mid‐pregnancy dietary scale consists of 13 items, including five core questions and eight general questions. The questionnaire encompassed all the suggested food groups and intake levels outlined in the Chinese Dietary Guidelines for Mid‐Pregnancy. For each item, the percentage of food in the dietary tower was used as the score. More details of this scale could be found in other studies (Ding et al. 2021). Generally, higher index scores were associated with a greater proportion of pregnant women consuming all types of food within the recommended range, while simultaneously indicating a lower proportion of those consuming less than the recommended range. Based on the whole score of the scale, it was possible to understand the difference between the dietary intake of pregnant women and the national dietary recommendations, as well as evaluate the general quality of a pregnant woman's diet at mid‐pregnancy. Specific scoring criteria (e.g., component weights, categorization) can be found in Tables S1 and S2.

### 2.8 Statistical Analysis

Statistical analyses were performed using IBM SPSS Statistics 24.0 and AMOS 24.0. Confirmatory factor analysis (CFA) was first conducted on the full sample to test the original four‐factor model of IES‐2 (Rburckle 2012). The maximum likelihood estimation method was applied (Rburckle 2012). Initial CFA indicated poor model fit (CMIN/DF = 6.568, CFI = 0.664), prompting modifications based on modification indices (e.g., correlating error terms of similarly worded items) (Tylka and Kroon van Diest 2013). After removing six low‐loading items (<0.40), a revised CFA model was re‐evaluated.

Exploratory factor analysis (EFA) was then performed on subsample A (N = 290). The Kaiser–Meyer–Olkin (KMO = 0.818) and Bartlett's test (*χ* ^2^ = 3851.755, *p* < 0.001) confirmed data suitability (Williams et al. 2012). A six‐factor structure was extracted via scree plot and eigenvalues (>1.0) (Cattell 1966), retaining items with loadings >0.40 and no cross‐loading (Brown 2006). The final CFA on subsample B (N = 291) validated the six‐factor model (CMIN/DF = 1.756, CFI = 0.925, RMSEA = 0.037) using fit indices (TLI, RMSEA, CFI) (Brown 2006; Browne 1993; Byrne 2012; Yu 2002).

Internal consistency was assessed via Cronbach's alpha. Concurrent validity was tested through Pearson correlations between IES‐2 scores and diet quality, depression, anxiety, and stress scores. Linear regression analyzed associations between IES‐2 factors and diet quality (*α* = 0.05, two‐tailed).

## 3 Results

### 3.1 Participants

The socio‐demographics of the women are given in Table 2.

**TABLE 2:** Sample characteristics (N = 581).

	Total *n*/mean	(%)/±SD
Age (years old)	30	4.81
Gestational week	24.17	1.94
Nutritional guidance in early pregnancy		
No.	301	51.8
Yes, but no effect	12	2.1
Yes, but the average effect	97	16.7
Yes, and great effect	171	29.4
Dietary behaviors in mid‐pregnancy		
I will follow nutritional guidelines carefully and be able to change my eating habits	199	34.3
Although I work/eat cafeteria/can't follow nutritional guidelines exactly, I will add my own meals.	112	19.3
I have to work/eat in a cafeteria/can't follow nutritional guidelines fully, so I haven't changed my diet.	22	3.8
I'm more spontaneous and change diets as I think of them	106	18.2
I didn't change my diet.	142	24.5
The disease diagnosed in mid‐pregnancy		
Have	158	27.2
No	423	72.8
Constipation		
Have	327	56.3
No	254	43.7
COVID‐19(N = 547)		
Have	244	42.0
No	303	52.2

### 3.2 Initial Confirmatory Factor Analysis

**FIGURE 1:**
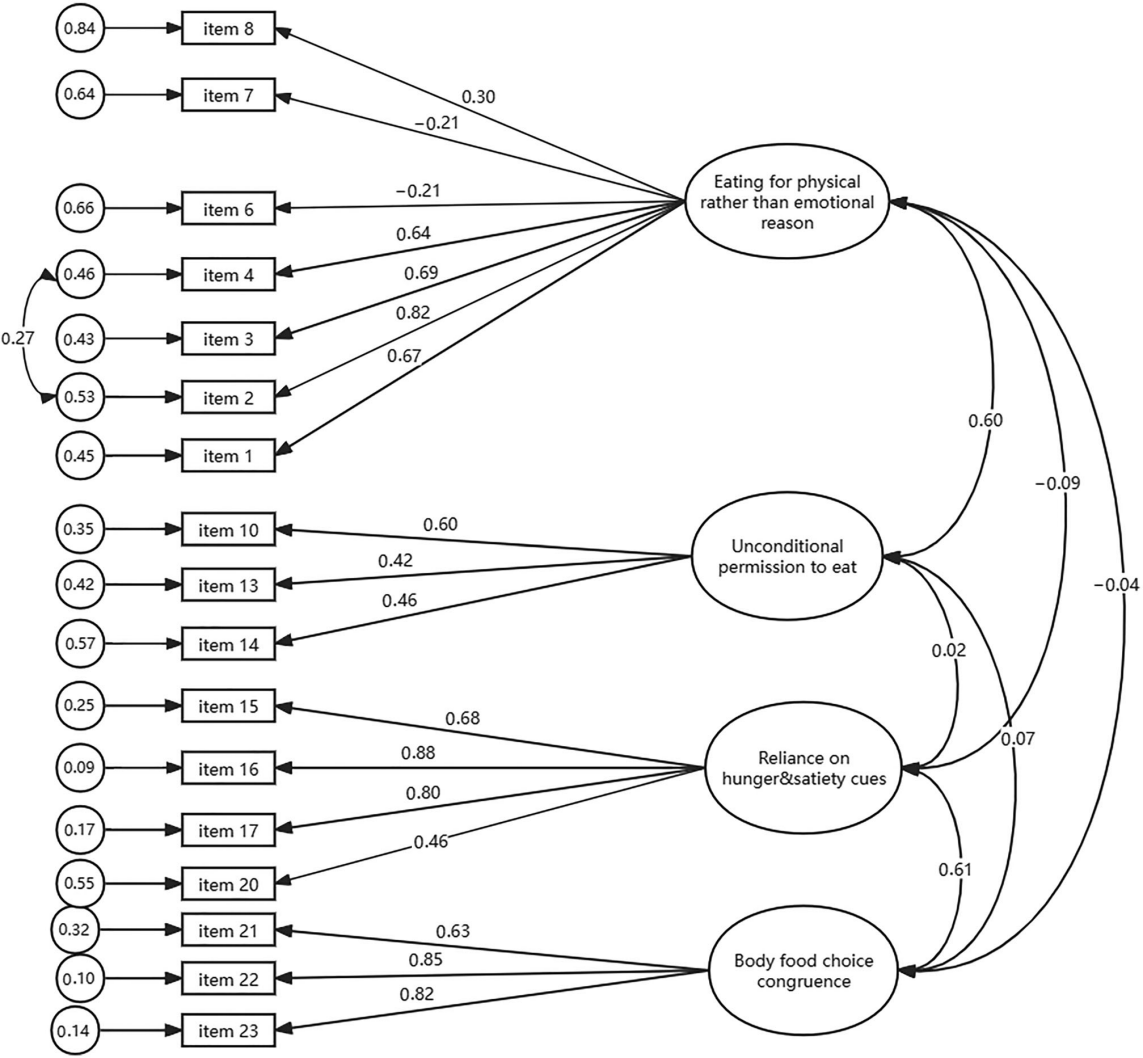
The initial confirmatory factor analysis of the original four‐factor structure of the IES‐2 scale.

Based on the results of the model fit statistics, the validated factor structure for the IES‐2 is not a good fit (CMIN/DF = 6.568, CFI = 0.664, TLI = 0.620, RMSEA = 0.097). There were 0.026–0.869 factor loadings for each indicator (i.e., item) variable, as shown in Table 3. As the initial model proved unsatisfactory parameters, several modifications were introduced. Based on modification indices, error variances of the items within the same factor were allowed to correlate (items 2 and 4). Because of the low loading value, six items were eliminated in the process. The details are shown in Table 4. Ultimately, we arrived at a more suitable model (CMIN (Chi‐square)/DF (degree of freedom) = 2.964, CFI (comparative fit index) = 0.927, TLI (Tucker‐Lewis index) = 0.911, RMSEA (Root mean square error of approximation) = 0.058) (Figure 1). Next, using the final model that contained 17 items and one error correlation, we performed a second‐order factor analysis. There was little indication that pregnant women had a second‐order IE factor because the residual variance other latent factor EPR in this second‐order model was negative (−1.04, p = 0.840). As a result, we were compelled to reject our last hypothesis that every latent component was a loading factor for the higher order IES factor.

**TABLE 3:** Confirmatory factor analysis factor loadings.

Items	Original factor	Estimate
1. I find myself eating when I'm feeling emotional, even when I'm not physically hungry.	F1	0.651
2. I find myself eating when I am lonely, even when I am not physically hungry.	F1	0.633
3. I use food to help me soothe my negative emotions.	F1	0.626
4. I find myself eating when I am stressed out, even when I'm not physically hungry.	F1	0.611
5. I am able to cope with my negative emotions without turning to food for comfort.	F1	−0.296^a^
6. When I am bored, I do not eat just for something to do.	F1	−0.376^a^
7. When I am lonely, I do not turn to food for comfort.	F1	−0.368^a^
8. I find other ways to cope with stress and anxiety than by eating.	F1	0.219^a^
9. I try to avoid certain foods high in fat, carbohydrates, or calories.	F2	−0.155^a^
10. If I am craving a certain food, I allow myself to have it.	F2	0.434
11. I get mad at myself for eating something unhealthy.	F2	0.033^a^
12. I have forbidden foods that I don't allow myself to eat.	F2	−0.078^a^
13. I allow myself to eat what food I desire at the moment.	F2	0.594
14. I do not follow eating rules or diet plans that dictate what, when and/or how much to eat.	F2	0.459
15. I trust my body to tell me when to eat.	F3	0.686
16. I trust my body to tell me what to eat.	F3	0.869
17. I trust my body to tell me how much to eat.	F3	0.810
18. I rely on my hunger signals to tell me when to eat.	F3	0.093^a^
19. I rely on my fullness signals to tell me when to stop eating.	F3	0.026^a^
20. I trust my body to tell me when to stop eating.	F3	0.466
21. Most of the time, I desire to eat nutritious foods.	F4	0.642
22. I mostly eat foods that make my body perform efficiently.	F4	0.852
23. I mostly eat foods that give my body energy and stamina.	F4	0.826
F1: Eating for physical rather than emotional reasons F2: Unconditional permission to eat F3: Reliance on hunger and satiety cues F4: Body–food choice congruence		

^a^ Loadings < 0.40

**TABLE 4:** Specifics of initial confirmatory factor analysis (CFA).

Operation	CMIN/DF	CFI	TLI	RMSEA
Delete item 5	4.318	0.808	0.77	0.07
Delete item 12	3.556	0.897	0.834	0.066
Delete item 9	3.148	0.888	0.869	0.061
Release residuals (items 2 and 4)	2.990	0.897	0.879	0.059
Delete item 11	3.000	0.907	0.889	0.059
Delete item 19	3.005	0.916	0.899	0.059
Delete item 18	2.964	0.927	0.911	0.058

### 3.3 Exploratory Factor Analysis

Initial EFA with varimax rotation with Kaiser normalization on sample A of IES‐2 (KMO  =  0.818; Bartlett's test *χ* ^2^ (290)  =  3851.755, *p* < 0.001) yielded a six‐factor structure with 56.794% of the total variance (Table 4). The loading was found below 0.50 in item 8 (“8. I find other ways to cope with stress and anxiety than by eating”), and item 12 (“12. ”I don't give myself to eat because I have forbidden foods). This model demonstrated an acceptable‐to‐good fit, with values of CMIN/DF = 2.475, CFI = 0.924, TLI = 0.909, and RMSEA = 0.050.

As a result of utility in scoring, we considered items 15–17 to be part of a single factor. In view of the stronger theoretical similarity between items 15, 16, 17 and the other items on factor 1 than there was between the other items in factor 6, it was decided to consider them as part of factor 1. In addition, items 15–17 were substantially more loaded on factor 1 than on factor 6. In the pretest, we learned from the interviews with pregnant women that they had a “cautious eating” attitude toward 15–17 items because of the word description “should.” Some of the pregnant women remarked: “I don't want to eat anything, but I must to eat anything,” “I don't like to eat something, I haven't eaten anything,” “I don't think these are safe and healthy enough to eat,” “ I still have serious vomiting, I don't know what to eat,” and “I don't want to eat anything, but I know that I should pay attention to my food.” The last changed IES‐2 was characterized by the following six factors: avoiding forbidden foods (five items), avoiding emotional eating (four items), body–food choice congruence (three items), avoiding food‐related coping strategies (three products), permission to eat (three items), and reliance on hunger and satiety cues (two items). To illustrate the theory of intuitive eating as a collection of healthy eating behaviors, several factors were designated with the negative adverb “avoiding.” Based on the results of the analysis, Cronbach's alphas were calculated as follows: overall score 0.78, avoiding forbidden foods 0.77, avoiding emotional eating 0.75, body–food choice congruence 0.82, avoiding food‐related coping strategies 0.78, permission to eat 0.58, and reliance on hunger and satiety cues 0.55. For the purposes of this analysis, items from the unconditional permission to eat subscale of IES‐2 were divided into two factors (i.e., avoiding forbidden foods and permission to eat). It has been determined that the items of the IES‐2 Eating for Physical Rather Than Emotional Reasons subscale and the items of the IES‐2 avoiding emotional eating subscale have been separated into two categories, referred to as Avoiding Eating and avoiding food‐related coping strategies, respectively. The resultant factor loadings are listed in Table 5.

**TABLE 5:** Factor loadings for the modified IES‐2 (six‐factor structure).

	Factors					
	1 Avoiding forbidden foods	2 Avoiding emotional eating	3 Body–food choice congruence	4 Avoiding food‐related coping strategies	5 Permission to eat	6 Reliance on hunger and satiety cues
16. I trust my body to tell me what to eat.	**0.807**	−0.036	0.285	0.019	0.046	−0.012
17. I trust my body to tell me how much to eat.	**0.784**	−0.036	0.274	0.052	0.042	−0.118
15. I trust my body to tell me when to eat.	**0.772**	−0.035	0.111	0.119	0.038	−0.010
9. I try to avoid certain foods high in fat, carbohydrates, or calories.	**0.549**	0.036	0.032	0.219	−0.200	0.060
20. I trust my body to tell me when to stop eating.	**0.527**	−0.005	0.243	0.093	−0.035	0.096
3. I use food to help me soothe my negative emotions.	0.098	**0.727**	−0.047	−0.045	0.162	0.106
4. I find myself eating when I am stressed out, even when I'm not physically hungry.	−0.176	**0.726**	0.102	−0.087	0.019	−0.163
2. I find myself eating when I am lonely, even when I am not physically hungry.	−0.119	**0.706**	0.040	−0.191	0.016	−0.089
1. I find myself eating when I'm feeling emotional, even when I'm not physically hungry.	0.098	**0.700**	−0.128	−0.112	0.132	0.255
8. I find other ways to cope with stress and anxiety than by eating.	0.047	0.382	0.205	0.214	0.129	0.051
22. I mostly eat foods that make my body perform efficiently.	0.317	−0.034	**0.753**	0.176	−0.059	0.108
21. Most of the time, I desire to eat nutritious foods.	0.232	0.114	**0.747**	0.066	0.136	−0.012
23. I mostly eat foods that give my body energy and stamina.	0.298	0.008	**0.740**	0.158	−0.039	0.228
12. I have forbidden foods that I don't allow myself to eat.	0.364	0.104	0.461	0.162	−0.160	0.004
7. When I am lonely, I do not turn to food for comfort.	0.148	−0.087	0.166	**0.860**	−0.042	−0.019
6. When I am bored, I do not eat just for something to do.	0.161	−0.086	0.032	**0.846**	−0.134	0.014
5. I am able to cope with my negative emotions without turning to food for comfort.	0.149	−0.131	0.286	**0.645**	0.208	−0.115
10.If I am craving a certain food, I allow myself to have it.	0.098	0.192	−0.111	0.061	**0.616**	0.402
14. I do not follow eating rules or diet plans that dictate what, when, and/or how much to eat.	−0.101	0.264	0.036	0.044	**0.592**	−0.236
13. I allow myself to eat what food I desire at the moment.	−0.012	0.337	0.115	−0.047	**0.533**	0.051
11. I get mad at myself for eating something unhealthy.	0.090	0.346	0.096	0.087	**−0.502**	0.112
18. I rely on my hunger signals to tell me when to eat.	0.082	0.080	0.024	−0.029	−0.192	**0.725**
19. I rely on my fullness signals to tell me when to stop eating.	−0.124	−0.026	0.254	−0.050	0.154	**0.646**

*Note*: Bold font indicates items loadings higher than 0.500.

### 3.4 Confirmatory Factor Analysis

**FIGURE 2:**
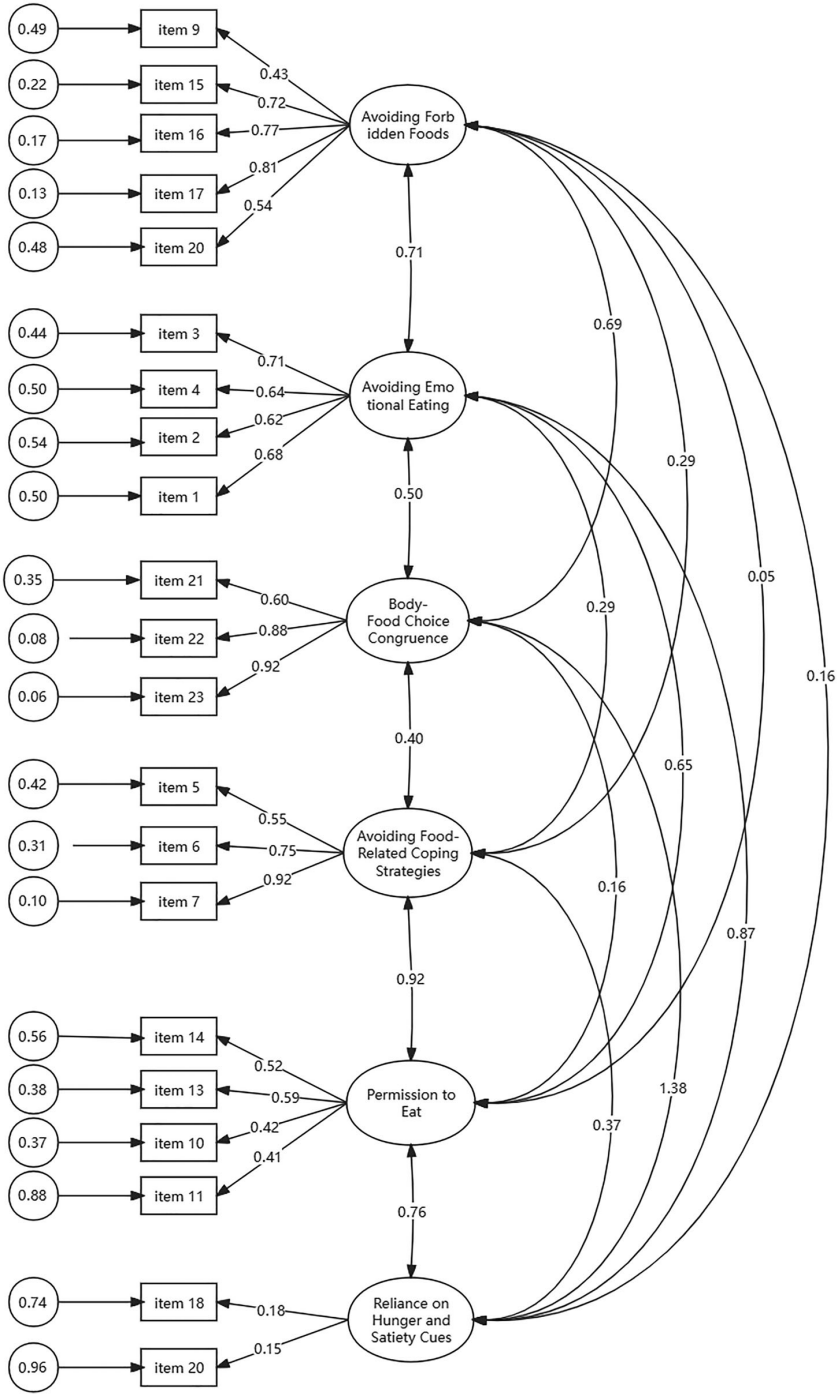
Standardized coefficients of confirmatory factor analysis results for a six‐factor model of the IES‐2 (modified).

On the basis of the EFA analysis, CFA was then conducted to examine a possible model of the IES‐2 in sample B (N = 291). Compared to the four‐factor models, the six‐factor model showed a better fit with a CMIN/DF of 1.756, a CFI of 0.925, a TLI of 0.909, and an RMSEA of 0.037. As a result, the six‐factor (avoiding forbidden foods [F1], avoiding emotional eating [F2], body–food choice congruence [F3], avoiding food‐related coping strategies [F4], permission to eat [F5], and reliance on hunger and satiety cues [F6]) the revised IES‐2 structure was verified and established for additional analysis (Figure 2).

### 3.5 Concurrent Validity

The IES‐2 (modified) model was found to be the best fit for our data; therefore, correlations were computed between the six subscales, total score, week, depression score, anxiety score, stress score, and diet quality (Table 6). Higher intuitive eating total scores were significantly correlated with higher diet scores (more appropriate eating), and factors scores were significantly correlated to different degrees, with anxiety, stress, depression, and diet quality, but not week.

**TABLE 6:** Correlations between IES‐2 (modified) and other variables.

	Anxiety score	Stress score	Depression score	Week	Diet quality
IES‐2 total scores	0.059	−0.069	−0.060	−0.043	0.209^**^
IES factor 1	−0.011	−0.199^**^	−0.208^**^	−0.071	0.316^**^
IES factor 2	0.065	0.159^**^	0.191^**^	0.027	0.105^*^
IES factor 3	0.050	−0.104^*^	−0.139^**^	−0.046	0.254^**^
IES factor 4	−0.008	−0.169^**^	−0.192^**^	−0.048	0.179^**^
IES factor 5	−0.017	0.044	0.053	−0.005	−0.004
IES factor 6	0.123^**^	0.070	0.127^**^	0.019	−0.020

*p < 0.05; **p < 0.01; ***p < 0.001.

### 3.6 Association of Intuitive Eating With Diet Quality in Pregnancy

We further investigated the association of IES‐2 (modified) with dietary quality in mid‐pregnancy. The results illustrated that for every one‐point increase in factors 1, 2, and 3, respectively, the dietary quality scores increased by 0.843, 0.312, and 0.690 points (*p* < 0.05) (Table 7).

**TABLE 7:** Regression prediction model of IES factors on diet quality.

	B	*t*	*p*	Covariance statistics	
				Tolerances	VIF
IES factor 1	0.843	4.983	0.000	0.684	1.463
IES factor 2	0.312	−2.088	0.037	0.799	1.251
IES factor 3	0.690	2.593	0.010	0.655	1.527
IES factor 4	0.131	0.646	0.519	0.780	1.282
IES factor 5	0.211	0.865	0.387	0.848	1.180
IES factor 6	−0.319	−1.236	0.217	0.956	1.046

## 4 Discussion

This study investigated the factor structure and psychometric features of the Chinese version of the IES‐2 scale in a group of pregnant women and further explored the relationship between intuitive eating and diet quality. This marks the first time the Chinese version of the IES‐2 scale has been utilized with a group of pregnant women, and the scale has been modified to accommodate the physiological changes associated with pregnancy. The psychological structure and reliability of the IES‐2 were also assessed in a group of pregnant women. There have been similar studies conducted in the past with groups of pregnant women, that is, with adaptations to the scale, but these studies were limited to the first edition of the scale (Daundasekara et al. 2017; Paterson et al. 2018).

First, this was confirmed using preliminary CFA methods: After deleting the six entries of the original intuitive diet, the deleted IES‐2 scale could also be applied to the group of pregnant women, but second‐order modeling was not possible. The details are as follows:

5. I am able to deal with my negative feelings without turning to food for comfort.

9. I make an effort to steer clear of certain foods that are high in fat, carbohydrates, or calories.

11. I get mad at myself for eating something unhealthy.

12. I don't give myself to eat because I don't have to do it because of my food.

18. When I want to eat, I use my hunger signals to tell me.

19. I use my fullness signals to indicate when to stop eating, and I use my wholeness signals as a way to tell me when to start eating.

At this point, the four subscales needed to be assessed separately. While this result differs from that of college students, previous studies of pregnant women using the IES scale have confirmed that this is the case (Daundasekara et al. 2017). In many past studies, entry deletion has been performed to varying degrees in the CFA validation of many studies (Babbott et al. 2022; Małachowska et al. 2021; Tylka et al. 2024). Considering that too many entries were deleted, there would be a decline in the validity of the scale, and it might not reflect certain factors. We found in our study that deleting entries from the four‐factor structures selected, as compared to retaining the original items, resulted in the next factor not being displayed, “Correspondence strategies related to food avoidance,” that is, deletion of the subsequent factor structures. Therefore, to better assess the intuitive eating of pregnant women, we divided the sample into two parts. We divided the sample into two parts for EFA and CFA cross‐validation to explore different factor structures adapted to the group of pregnant women.

Our study established a six‐factor structure. In the six‐factor structure, we deleted the following two items:

8. When compared to eating, I have more methods to deal with stress and anxiety than eating.

12. Don't give myself to eat because I don't have to do it because of my food.

The structures used here were quite similar to those of earlier research (Khalsa et al. 2019; Madanat et al. 2020). After excluding two items with insufficient loading, the “body–food choice congruence” was unchanged in the modified scale compared to the original scale. Previous research has shown similar results (Khalsa et al. 2019; van Dyck et al. 2016). The fact that this scale has a higher degree of stability in terms of component structure is plausible. Thus, some studies selected only this subscale in the IES to assess intuitive eating behavior (Małachowska et al. 2023).

In our study, “Unconditional permission to eat” was categorized as avoiding eating and allowing eating. According to the original description of intuitive eating, the ability to eat without any rules or conditions was considered an important aspect of intuitive eating as people who allowed themselves to eat (rather than forbidding themselves from eating) avoided subsequent food obsessions and binge eating. Again, in our study, these concepts actually represent two separate aspects of intuitive eating. The results of previous research conducted in different cultures showed that pregnant women spontaneously reduced restrictive eating habits and gave themselves greater permission to eat (Paterson et al. 2018). Compared to pre‐pregnancy, avoiding foods higher in calories, fats, or carbohydrates is reduced and guilt is diminished (Paterson et al. 2018). Additionally, permissive eating on the UPE scale is associated with permissive behaviors, such as craving, which is a common and well‐recognized behavior among pregnant women (Forbes et al. 2018). The results of this study were not observed in previous studies conducted on pre‐pregnant women or a sample of regular female university students (Daundasekara et al. 2017; Tylka and Kroon van Diest 2013). Consequently, pregnancy may cause pregnant women to change their eating habits, such as avoiding food and dividing food. Additionally, another study among Mexican pregnant women, which included pregnant women at various stages of their pregnancy (early‐to‐late pregnancy), found that the subscale was not useful (Flores‐Quijano et al. 2023). We hypothesized that this could be attributed to a pregnancy‐related effect. Pregnant women may not be as aware of their pregnancy during pre‐pregnancy as they are during mid‐pregnancy, so they behave dietary as if they are not pregnant. It is anticipated that the subjective awareness of pregnancy will become stronger in mid‐to‐late pregnancy and that eating behaviors will change accordingly. For example, depression and anxiety symptoms are much more prevalent during mid‐to‐late pregnancy than during early pregnancy (Heron et al. 2004). Therefore, observing pregnant women in early and late pregnancy at the same time may result in indistinct results and thus be insignificant. We limited our study population to mid‐pregnancy, so future studies may examine different stages of pregnancy.

Furthermore, the items within the “reliance on hunger and satiety cues” subscale were categorized into two factors: “avoiding emotional eating” and “avoiding food‐related coping strategies.” This division arises because items pertaining to eating in response to emotional states such as loneliness, stress, or negative feelings cluster together in one factor (avoiding emotional eating). Conversely, the items that specifically mention using food as a means of coping or seeking comfort fall under the second factor (avoiding food‐related coping strategies), thus highlighting two separate dimensions of intuitive eating. Eating in reaction to physical hunger and satiety was kept for some of the original items. In contrast to previous studies, the three items originally belonging to “reliance on hunger and satiety cues” were loaded on the “avoiding forbidden foods.” In this regard, we found that the entries all included “my body will tell me … I need to…”, and in the pretest interviews, respondents indicated a “cautious eating” attitude toward them. This is reflected in previous IES interviews with pregnant women (Paterson et al. 2018). Many of the pregnant women indicated that they had trouble eating because of vomiting reaction. They reported not feeling hungry, and not wanting to eat. Even though they realized they should eat, they ultimately chose to avoid eating to reduce the likelihood of vomiting. As a result, this might be a unique feature of the group of pregnant women. There are two problems which do not load on any single factor. This sort of makes sense because pregnant women have a very strong reason (their babies) to be concerned with what they eat for the health of their pregnancy and babies. Perhaps “unconditional permission to eat” in general is unrealistic for pregnant women and may should not be considered healthy since there are certain dietary practices that could be unsafe for pregnant women (consumption of certain cheeses and fish).

In addition to the association between consumable eating scores and the total amount of food that expectant mothers eat, our research also shows how these scores are related to the general nutritional quality of expectant moms. The same outcomes as in a prior investigation were obtained. Specifically, we found that pregnant women with higher scores on intuitive eating tend to have better dietary quality (Małachowska et al. 2023). The Chinese Dietary Guidelines Adherence Score for Pregnant Women developed and evaluated by Prof. Wang Zhixu and his research group members serves as a reliable and simple method for measuring the adherence of pregnant women to the dietary principles described in the Chinese Dietary Guidelines (2016). This tool has been widely used in primary pregnancy nutrition clinics (Ding et al. 2021). A higher score indicates a higher proportion of pregnant women consuming each type of food within the recommended range and a lower proportion falling below the recommended range. Pregnant women with higher intuition scores had healthier diet quality. Previous studies rarely assessed the overall diet quality of pregnant women, and Jessica et al. found a correlation between the intuitive eating score and the consumption of vegetables and fruit (Saunders et al. 2018). Furthermore, one study found a cross‐sectional and longitudinal 1‐year association between intuitive eating and improved diet quality in women with gestational diabetes (Quansah et al. 2022). It should be noted that a single food evaluation is not sufficient to support the overall quality of dietary intake. As an example, a pregnant woman may choose to eat a lot of fruit (rather than desserts because fruit is a “healthy food”) to relieve her depression. The consumption of large quantities of fruit during pregnancy can cause an increase in blood glucose levels, which can lead to gestational diabetes (Zhang et al. 2006). The positive correlation between intuitive eating and dietary quality suggests that it may improve metabolic health during pregnancy through mechanisms such as reducing emotional eating and avoiding forbidden foods. For example, some researchers have found that intuitive eating behaviors reduce glycemic fluctuations in pregnant women with gestational diabetes (Gao et al. 2024). In the future, intuitive eating principles can be integrated into prenatal nutrition education, and their role in weight management and complication prevention during pregnancy can be validated through intervention studies.

In addition, the low Cronbach's *α* values (0.58 and 0.55) for Factor 5 (permission to eat) and Factor 6 (reliance on hunger and satiety cues) in the present study might be attributed to the following reasons: (1) number of items limitation: Factor 6 contain only two items, and the smaller number of entries tends to reduce internal consistency; (2) physiological disorders during pregnancy: Prior interviews showed that pregnant women had doubts about the existence of topics related to the reaction to pregnancy vomiting, which is directly related to questions 15–17. This physiological and psychological conflict leads to the possibility that the formulation of the relevant entries may need to be further adapted to better fit the experience of pregnancy; (3) cultural specificity: for example, entry 14 (“I don't follow dietary rules”) may trigger contradictory interpretations in Chinese culture, which emphasizes “dietary safety during pregnancy,” and some pregnant women chose to answer conservatively. Also, in traditional Chinese thinking, older mothers believe that “only poor people eat less” and therefore “the more you eat, the better you will be.” Differences between the six‐factor structure of the present study and the five‐factor model for Mexican pregnant women may stem from different cultural understandings of “unconditional permission to eat.” Chinese pregnant women were more likely to distinguish between “permission to eat” and “Avoidance Forbidden Foods” as independent dimensions, reflecting their concern for dietary safety during pregnancy (Flores‐Quijano et al. 2023).

Our study showed that the subscales were significantly and negatively associated with depression, anxiety, and pregnancy stress during pregnancy. This indicates that pregnant women who exhibit higher IES‐related eating behaviors are more likely to experience lower symptoms of depression, anxiety, and stress. According to previous studies showing that adaptive eating behaviors are related to improved psychological status (Dockendorff et al. 2012), this result is in accordance with eating behavior theory. Female self‐compassion was considered a mediating factor in mediating eating according to Carbonneau et al. (2021). Thus, the psychological state is also a key factor influencing eating behavior during pregnancy, but current research could hardly determine a causal association between the two.

The limitations of this study should be acknowledged. First, we did not investigate the actual dietary frequency of pregnant women, so there might be a bias when compared with the actual situation. Second, we did not collect data before pregnancy. Pregnant women's dietary habits differ greatly throughout pregnancy, and the physiological and psychological changes that occur during pregnancies and early pregnancy may obscure the effects in mid‐pregnancy. Research on pregnant women's diets should consider distinguishing between gestational stages rather than relying solely on the overall gestational period in the future. The samples in this study were all from a single center in Danyang City, Jiangsu Province, which may have regional cultural bias. Future studies need to include multi‐regional and multi‐cultural groups of pregnant women to verify the generalizability of the results.

## 5 Conclusion

According to our study, the modified six factors might be more appropriate for assessing intuitive eating in pregnant women with the IES‐2 scale. It is more likely that the higher her intuitive eating score is, the better her diet is. There is a need for a better understanding of the structure of intuitive eating in special populations in light of the current findings. It is possible that intuitive eating behaviors could be interpreted differently under different physiological conditions. Pregnant women, particularly those in the early and middle stages of pregnancy, may experience a change in their eating patterns and eating habits because of pregnancy reactions. To promote maternal and infant health, more research is required to examine the relationship between intuitive eating and health during pregnancy in pregnant women eating.

## Author Contributions

**Xingyi Jin**: conceptualization, writing–original draft. **Jian Zhu**: funding acquisition, investigation. **Da Pan**: writing–review and editing. **Lingzhen Sun**: investigation. **Rui Wang**: formal analysis. **Niannian Wang**: visualization. **Jiongnan Wang**: software. **Chunyan Yuan**: project administration. **Shaokang Wang**: supervision, resources. **Guiju Sun**: supervision, resources.

## Ethics Statement

The study was carried out in accordance with the Hel‐sinki Declaration principles and was approved by the Danyang Maternal and Child Health Hospital (15/9/2022). Informed consent was obtained from all subjects involved in the study.

## Conflicts of Interest

The authors declare no conflict of interest.

### Peer Review

The peer review history for this article is available at https://publons.com/publon/10.1002/brb3.70568

## Supporting information



Supplementary Information

## Data Availability

The data that support the findings of this study are available on request from the corresponding author. The data are not publicly available due to privacy or ethical restrictions.
