# Supplementary material for: The Chinese Version of the Intuitive Eating Scale‐2 Adapted for Pregnant Women: Psychometric Properties and Associations With Diet Quality
Source: Brain Behav. 2025 May 30;15(6):e70568. doi: 10.1002/brb3.70568 (PMC12123098; doi:10.1002/brb3.70568)
Supplement: Supplementary file 1 — Supplementary Information [file BRB3-15-e70568-s001.docx]

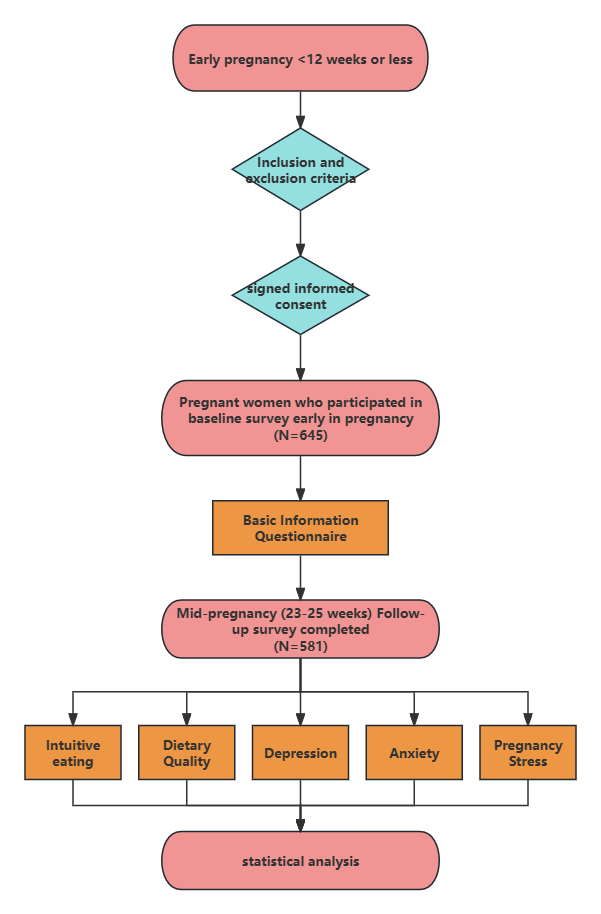


**Figure S1. Flowchart**

**Components of CDGCI-PW**

Since this study only assessed diet quality in the second trimester, only the CDGCI-PW in the second trimester was presented (Table S1). Components 1 and 2 measured whether the amount of variety in a person’s diet meets the recommendations of diversification specified in the CDG-PW. Components 3 to 12 measured the degree to which a person’s diet aligns with the recommendations of the CDG-PW for nine major food groups: staple food (cereals and their products, potatoes, and beans other than soybeans); whole grains and beans other than soybeans; green leafy and colored vegetables (red and yellow); milk and its products; soybean and its products; nuts; lean meat (livestock and poultry meat); animal blood and liver; and iodized table salt and iodine-rich seafood. Unhealthy diets, such as those including excessive intake of fat, sugar, and salt, may have short- and long-term health effects on pregnant women and their fetuses. Therefore, we included component 13 to measure a person’s healthy eating habits.

**Table S1.**Key recommendations of dietary guidelines for pregnant women and components of CDGCI-PW.

| **Key Recommendation** | **Components of CDGCI-PW** |
| --- | --- |
| (1) Eat a variety of foods, mainly cereals and their products (a balanced diet includes 11 categories of foods per week and 12 kinds of foods per day. Whole grains and beans other than soybeans should form no less than one-third of the total intake of staple food). | 1. How many categories of food do you eat per week on average? |
|  | 2. How many kinds of foods do you eat per day on average? |
|  | 3. Can whole grains and beans other than soybeans account for more than one-third of your staple food intake? |
| (2) Eat a balanced diet with no less than 130 g of carbohydrates per day. | 4. Does your daily intake of staple food reach 200 g on average? |
| (3) Ensure adequate intake of vegetables, milk and its products, soybeans and its products, and nuts. Among them, the intake of green leafy and colored vegetables (red and yellow) should reach 200 g per day. | 5. Do you eat more than 200 g of green leafy and colored vegetables (red and yellow) per day on average (raw weight)? |
|  | 6. How often do you drink milk and its products per week on average? Dairy intake is considered to be significant when servings are equivalent to 250 mL of fresh liquid milk each time. |
|  | 7. How often do you eat soybeans and soybean products per week on average? The intake of soybeans products is considered to be significant when servings are the equivalent of up to 15 g of dry soybeans each time. |
|  | 8. How often do you eat nuts per week on average? Nuts intake is considered to be significant when servings are the equivalent of up to 10 g of dry nuts each time. |
| (4) Eat appropriate amounts of lean meat (livestock and poultry meat), aquatic products (fish, shrimp, and shellfish), and eggs. Eat iron-rich foods such as animal blood or liver once or twice a week. | 9. Do you eat 125 g of lean meat (livestock and poultry meat), aquatic products (fish, shrimp, and shellfish), or eggs per day on average? |
|  | 10. How often do you eat animal blood and liver per week on average? The intake of animal blood and liver is considered to be significant when it is reach to 20 to 50 g each time. |
| (5) Eat iodized table salt and iodine-rich seafood. | 11. Do you eat iodized table salt every day? |
|  | 12. How often do you eat iodine-rich seafood per week on average? This includes kelp, nori, undaria pinnatifida, shellfish, sea fish, etc. |
| (6) Develop healthy eating habits | 13. Do you often eat foods high in oil, salt, and sugar? |

CDGCI-PW: Chinese dietary guidelines compliance index for pregnant women.

**Scoring Criteria for CDGCI-PW**

Each component was assigned a different score range, and the overall index ranged from 0 to 100 points. Details of the scoring criteria are shown in Table S2. Food variety was the basic principle of a balanced diet. According to the dietary guidelines for Chinese residents, a balanced diet includes 11 categories of food per week and 12 kinds of food per day. Therefore, components 1 and 2 were of great importance; with scores ranging from 0 to 25 points, they accounted for a quarter of the total score. Components 3 to 12 were all scored based on whether the intake of food met the minimum recommendations of the Chinese balanced dietary pagoda for pregnant women. The weights of components 3, 4, 6, 7, 8, and 11 were the same, with scores ranging from 0 to 4 points. According to CDG-PW, pregnant women should consume iron-rich foods, iodized table salt, ensure the intake of necessary carbohydrates, and appropriately increase the intake of lean livestock meat, poultry, aquatic products (fish, shrimp, and shellfish), eggs, milk and its products. Therefore, the weights of components 5, 9, 10, and 12 were increased, with a maximum score of five points. Component 13 measured poor eating habits, involving pickled food, fried food, cream cake, chocolate, and other high-salt, high-oil, and high-sugar food. The consumption of these foods more than three times per week on average indicated poor eating habits and scored as 0 points; otherwise, the score was 6 points.

**Table S2.** Scoring criterion of CDGCI-PW.

| **Components** | **Scoring Criterion** | **Range of Score** |
| --- | --- | --- |
| 1. How many categories of food do you eat per week on average? | A. Less than or equal to 6 categories | 0 points |
|  | B. Range from 7 to 10 categories | 5 to 20 points (5 points for each additional category) |
|  | C. More than or equal to 11 categories | 25 points |
| 2. How many types of foods do you eat per day on average? | A. Less than or equal to 4 kinds | 0 points |
|  | B. Range from 5 to 11 kinds | 1 to 7 points (1 point for each additional kind) |
|  | C. Range from 11 to 20 kinds | 7 to 25 points (2 points for each additional kind) |
|  | D. More than or equal to 20 kinds | 25 points |
| 3. Can whole grains and beans other than soybeans account for more than one-third of your staple food intake? | A. No | 0 points |
|  | B. Yes | 4 points |
| 4. Does your daily intake of staple food reach 200 g on average? | A. No | 0 points |
|  | B. Yes | 4 points |
| 5. Do you eat more than 200 g of green leafy and colored vegetables (red and yellow) per day on average? (raw weight) | A. No | 0 points |
|  | B. Yes | 5 points |
| 6. How often do you drink milk and its products per week on average? Dairy intake is considered to be significant when servings are equivalent to 250 mL of fresh liquid milk each time. | A. Less than or equal to once a week | 0 points |
|  | B. Range from 2 to 4 times per week | 1 to 3 points (1 point for each additional time per week) |
|  | C. More than or equal to 5 times per week | 4 points |
| 7. How often do you eat soybeans and soybean products per week on average? The intake of soybeans products is considered to be significant when servings are the equivalent of up to 15 g of dry soybeans each time. | A. Less than or equal to once a week | 0 points |
|  | B. Range from 2 to 4 times per week | 1 to 3 points (1 point for each additional time per week) |
|  | C. More than or equal to 5 times per week | 4 points |
| 8. How often do you eat nuts per week on average? Nuts intake is considered to be significant when servings are the equivalent of up to 10 g of dry nuts each time. | A. Less than or equal to once a week | 0 points |
|  | B. Range from 2 to 4 times per week | 1 to 3 points (1 point for each additional time per week) |
|  | C. More than or equal to 5 times per week | 4 points |
| 9. Do you eat 125 g of lean meat (livestock and poultry meat), aquatic products (fish, shrimp, and shellfish), or eggs per day on average? | A. No | 0 points |
|  | B. Yes | 5 points |
| 10. How often do you eat animal blood and liver per week on average? The intake of animal blood and liver is considered to be significant when it reaches 20 to 50 g each serving. | A. Never | 0 points |
|  | B. 1 time per week | 3 points |
|  | C. More than or equal to 2 times per week | 5 points |
| 11. Do you eat iodized table salt every day? | A. No | 0 points |
|  | B. Yes | 4 points |
| 12. How often do you eat iodine-rich seafood per week on average? This includes kelp, nori, undaria pinnatifida, shellfish, sea fish, etc. | A. Never | 0 points |
|  | B. Range from 1 to 4 times per week | 1 to 4 points (1 point for each additional time per week) |
|  | C. More than or equal to 5 times per week | 5 points |
| 13. Do you often eat foods high in oil, salt and sugar? | A. Yes | 0 points |
|  | B. No | 6 points |

CDGCI-PW, Chinese dietary guidelines compliance index for pregnant women.
